# Surgical Management of Radial Head and Proximal Ulna Fractures Without Elbow Dislocation: A Case Report

**DOI:** 10.7759/cureus.74987

**Published:** 2024-12-02

**Authors:** Yahya Deniz, Arın Celayir, Hasan Marangoz, Mehmet Fatih Guven

**Affiliations:** 1 Orthopedics and Traumatology, Istanbul University-Cerrahpasa, Istanbul, TUR

**Keywords:** distal radioulnar joint (druj), monteggia's fracture, proximal ulna fracture, radial head fracture, radial head prosthesis

## Abstract

Fractures involving the proximal ulna and radial head are common injuries that occur in the upper extremity, often resulting from traumatic incidents such as falls or direct impact. The proximal ulna forms the elbow joint with the humerus, while the radial head articulates with both the humerus and the ulna, facilitating forearm rotation. Fractures in these areas can disrupt the stability and function of the elbow joint, leading to pain, swelling, and limited range of motion.

Clinically, it is more common to observe a radial head dislocation with a proximal ulna fracture. This fracture is referred to as a Monteggia fracture-dislocation. A radial head fracture and proximal ulna fracture occurring independently are not frequently encountered. In this study, we aim to discuss the surgical intervention performed on a patient with fractures of the proximal ulna and radial head, as well as the postoperative physical therapy follow-up.

## Introduction

Fractures affecting the proximal ulna and radial head are frequent injuries in the upper limb, often stemming from traumatic events like falls or direct impacts. The proximal ulna contributes to the elbow joint's formation with the humerus, while the radial head enables forearm rotation by articulating with both the humerus and ulna [1]. Such fractures can disturb elbow joint stability and function, leading to symptoms like pain, swelling, and restricted movement [2].

Fractures of the radius and ulna represent the most prevalent fractures of the upper extremity, with distal fractures occurring more frequently than proximal ones. These fractures often result from falls onto outstretched hands, constituting the primary mechanism of injury. Diagnosis is typically confirmed through radiography or ultrasonography evaluation [3].

Monteggia fracture-dislocations are serious injuries characterized by a fracture of the ulna shaft coupled with a dislocation of the radial head at the elbow. This type of injury typically results from a fall on an outstretched hand or direct trauma to the forearm. The Bado classification system categorizes Monteggia fractures into four types based on the direction of radial head dislocation and the nature of the ulnar fracture. Type I involves the anterior dislocation of the radial head, type II posterior, type III lateral, and type IV includes both an ulnar and radial shaft fracture with radial head dislocation [4]. Our patient's fracture also could not be classified under the Monteggia classification. The fracture pattern in our patient does not conform to the Monteggia fracture-dislocation type. After the ulna fracture, it was assessed as a compression fracture extending to the metaphysis of the radial head, caused by axial loading on the radius. However, isolated occurrences of radial head fractures or proximal ulna fractures are relatively uncommon.

## Case presentation

**Figure 1:**
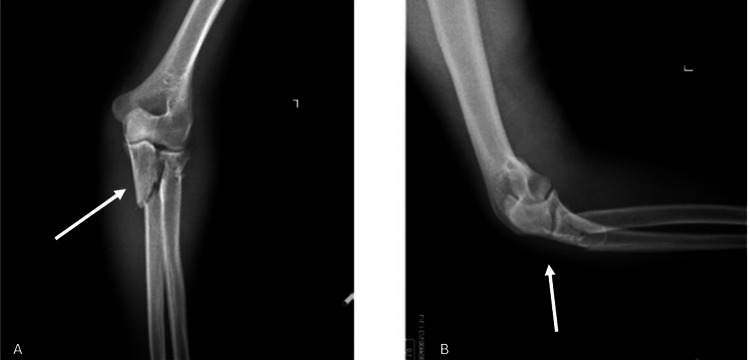
X-ray images of the patient at hospital admission. Both anteroposterior (A) and lateral (B) X-ray images confirm a fracture at proximal ulna and radius White arrows indicate the fracture site at proximal ulna and radius.

A 33-year-old right-handed male tennis player presented to our department after a fall from a motorcycle, with his wrist in dorsiflexion and elbow in extension. Upon evaluation, proximal ulna and radial head fractures without dislocation were identified on his left side (Figure 1). However, our patient's fracture could not be classified under the Monteggia classification. In this patient, the fracture of the ulna caused a compression-type fracture in the radial head, leading to the progression of this fracture into the metaphyseal region.

**Figure 2:**
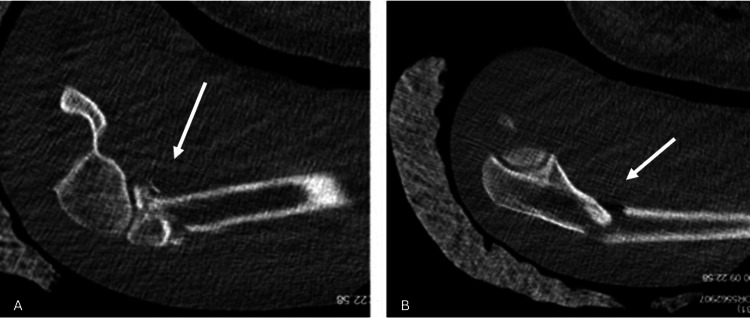
Sagittal section of the computed tomography images of the patient. Radial head fracture with subluxation (A) and proximal ulna fracture (B) can be seen in the tomography sections White arrows indicate the fracture at the proximal ulna.

**Figure 3:**
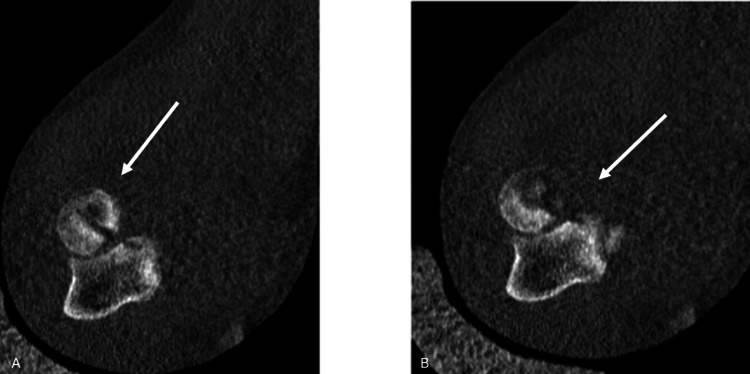
Axial section of the computed tomography images of the patient. Both A and B demonstrate the fracture in the radial head White arrows indicate the fracture at proximal radius.

**Figure 4:**
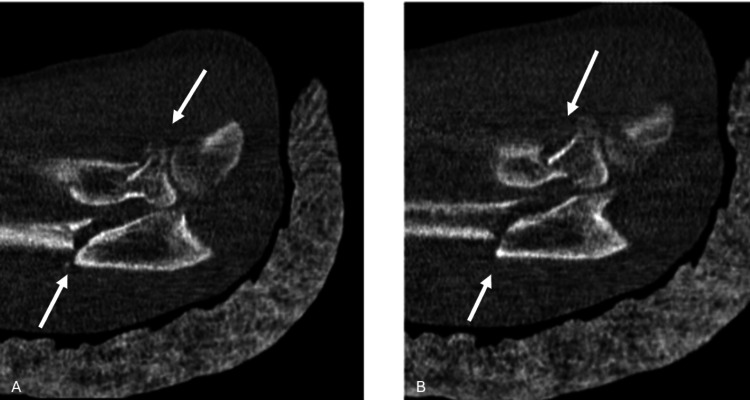
Coronal section of the computed tomography images of the patient. A and B demonstrate the fractures at the proximal ulna and radial head subluxation White arrows indicate the fractures at proximal ulna and radius.

The patient was fitted with a long arm splint, and preparations for surgery commenced. He was admitted to the ward. Informed consent was obtained from the patient before any medical procedures were performed. Elbow tomography was performed on the patient (Figures 2-4). The patient underwent surgery the following day.

The patient underwent surgery for a left proximal ulna fracture and a radial head fracture. A 15 cm longitudinal incision was made along the posterior aspect of the ulna, starting proximally from the olecranon and extending distally along the subcutaneous border. Care was taken to protect the surrounding soft tissue and neurovascular structures during the procedure. An anatomical olecranon plate was used for alignment, with five screws proximally and four screws distally for stabilization. A Kaplan incision was made for the radius head fracture, followed by fracture line curettage and stabilization with six screws via an anatomical radius proximal plate. Closure was done following anatomical guidelines, and a long arm splint was applied postoperatively.

**Figure 5:**
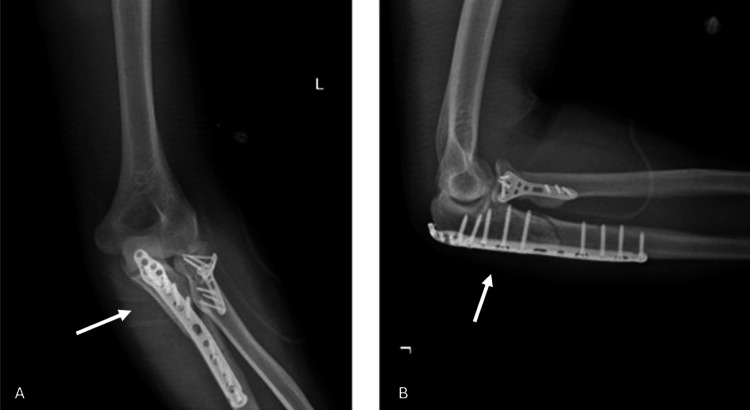
Postoperative X-ray images of the patient can be seen on the anteroposterior (A) and the lateral (B) view White arrows indicate the implant after surgery.

The patient was evaluated postoperatively (Figure 5), and examination revealed a clean wound without any discharge, and no vascular deficit was detected.

Upon discharge, the patient was prescribed endol and nonsteroidal anti-inflammatory drugs (NSAIDs). A routine outpatient clinic follow-up was scheduled two weeks after surgery. During the first follow-up, the splint was removed, and the patient transitioned to a shoulder-arm sling, and passive elbow flexion and extension exercises were initiated. At the second follow-up, four weeks postoperatively, the sling was maintained, and early-phase passive elbow flexion and extension exercises were continued, with the addition of pronation and supination movements. At the third follow-up, six weeks postoperatively, the sling was removed entirely. By the 1.5-month follow-up, the patient demonstrated 100 degrees of elbow flexion with a 15-degree loss of extension. The patient was subsequently referred for physical therapy rehabilitation to further improve joint function and range of motion.

**Figure 6:**
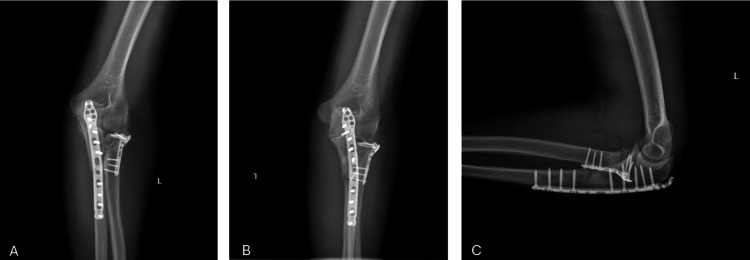
The patient's ninth-month follow-up radiographs: (A) anteroposterior (AP) view, (B) oblique view, and (C) lateral view are shown

**Figure 7:**
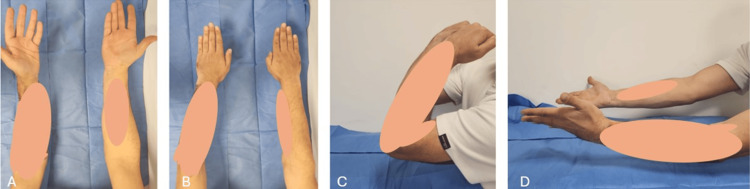
The patient's ninth-month follow-up physical examination findings: (A) a 10-degree loss of supination is observed; (B) pronation is observed to be complete; (C) flexion is observed to be complete; (D) supination restriction is noted

At the ninth-month follow-up, control radiographs were taken (Figure 6), and postoperative movements were evaluated (Figure 7).

It was observed that flexion and extension were full, pronation was complete, and supination was restricted by 15 degrees. The patient stated that they had returned to their sports activities and continued working as a personal trainer, expressing satisfaction with the outcome.

## Discussion

A Monteggia fracture-dislocation entails a fracture in the proximal one-third of the ulna accompanied by a dislocation of the radial head proximally at the elbow. This injury pattern typically occurs due to a low-energy mechanism, such as a fall on the outstretched hand [5].

In our patient, although the mechanism was similar, a complete radial head dislocation was not observed. Instead, a proximal ulna fracture was identified along with a radial head fracture and subluxation. During the preoperative evaluation, we planned for surgery using olecranon plates, proximal radius plates, and a radial head prosthesis.

During the operation, we first stabilized the ulna and then proceeded to address the radial head. We noticed that the radial head fracture was more fragmented and complicated than anticipated. Initially, we considered a prosthesis due to the fragmented nature of the fracture, but when we found that the fracture extended to the metaphysis, we opted for reconstruction and fixation with a plate. In such cases where there is excessive axial loading on the radial head due to the fracture of the proximal ulna, radial head fractures can be fragmented and complicated. Surgical planning should include both prosthesis and plate options.

The objective of any surgical procedure for proximal ulna and radial head fractures should prioritize early functional elbow rehabilitation while considering all mended structures. Initially, the elbow is immobilized in a plaster cast at a 90° flexion for approximately two weeks. However, depending on the condition of the soft tissues, it is advisable to initiate active and active-assisted early motion as early as day two or three post-surgery under the supervision of a physiotherapist. This approach aims to mitigate the risk of postoperative elbow stiffness [6].

In the study conducted by Siebenlist and colleagues, the importance of radiological evaluation at six weeks postoperatively for assessing fracture union is emphasized, and it is recommended that a return to sports should occur between the third and fifth months after surgery to prevent stiffness in the elbow [5]. Therefore, we decided to implement early passive elbow exercises and endol therapy to prevent stiffness in the elbow. We planned for our patient to return to tennis in a controlled manner in the third month postoperatively. As anticipated, the patient resumed playing tennis in the third postoperative month and continued their training as a personal trainer. At the ninth-month follow-up, the patient's elbow flexion and extension were fully intact, pronation was complete, but supination was limited by 15 degrees. However, the patient reported no restrictions in their sports activities and expressed satisfaction with the result.

Elbow stiffness caused by heterotopic ossification (HO) can significantly impair daily activities, threatening functional independence. HO is defined by the formation of mature lamellar bone in non-osseous tissues [6]. Although the mechanisms underlying periarticular HO are not fully understood, advancements in our knowledge of elbow anatomy and the pathophysiology of HO have facilitated early excision procedures, enabling faster functional recovery [7]. To prevent HO formation, we administered prophylactic endol therapy postoperatively, a critical measure to minimize the risk of functional impairment. The use of pharmacological agents such as NSAIDs, bisphosphonates, and radiotherapy, along with prophylactic endol therapy, is well supported in the literature. Board et al. [8] emphasized the importance of prophylactic strategies in preventing HO after lower limb arthroplasty, while Baird and Kang [9] highlighted early NSAID intervention as a key factor in reducing HO incidence and severity. By integrating these evidence-based approaches, including prophylactic endol therapy, into our protocol, we successfully optimized the patient's recovery and functional outcomes.

## Conclusions

In conclusion, the combination of a proximal ulna fracture and a fragmented radial head fracture presents a complex surgical challenge. In this case, plate fixation was selected as the definitive treatment for the radial head fracture, and the patient's excellent clinical recovery validates this approach. Despite the potential for incomplete radiological healing in such injuries, early initiation of movement plays a critical role in preventing functional limitations and promoting optimal recovery. This case highlights the significance of personalized surgical planning and rehabilitation strategies in achieving favorable outcomes for complex fracture patterns.
